# On Some Effects of Inflammation of the Membranous Lining of the Larynx, &c.

**Published:** 1833-04-01

**Authors:** 


### On some Effects of Inflammation of the Membranous Lining of the Larynx, &c. &c. By *John Wood*, late House Surgeon to St. Bartholomew's Hospital.

[Med. Chir. Trans. Vol. XVII.]

This is a very long paper, exhibiting very creditable research and much judgment on an important point of medical surgery. Laryngitis is not uncommon, and every one knows what a fearful and formidable inflammation it is. In many cases it comes on almost insensibly and proceeds slowly, and is not usually complicated with inflammation of the tracheal or bronchial lining, or with much symptomatic fever. It is attended with more or less dysphagia, by which our author thinks it may be distinguished from croup. Pathologically too, he avers that it differs from the latter disease, inasmuch as the change of structure consists of interstitial deposition, and not of effusion, in the form of an adventitious membrane upon the surface of the inflamed mucous membrane.

"The distinctions of croup from other forms of inflammation of the larynx, and an accurate knowledge of the seat of obstruction in each instance, are essential to successful treatment of the different affections. General bleeding, so highly beneficial in croup, seems often to have aggravated the sufferings, and hastened the death, of those affected with œdema of the glottis. Had the pathology of croup been better understood, laryngotomy would never have been declared to be the most efficacious means of relief." 142.

It is often impossible to determine, during life, whether acute inflammation has caused serous effusion into the submucous tissue, or whether this is swollen and thickened by gradual interstitial deposition.

"It appears to me that the term SPASM is often used unwarrantably to explain the deaths of persons who have shewn symptoms of diseased larynx, and that the common notion of spasmodic asphyxia is dangerous, as it leads to the use of opiates and antispasmodics in disorders essentially inflammatory, and only to be treated with success by those who early discover and appreciate their nature and tendency. Dissection, certainly seldom discloses in the larynx a complete obstacle to the transmission of air: this is not necessary to produce death. If the passage be gradually narrowed, so as to prevent at each inspiration the ingress of a proper quantity of air, the properties of the blood are consequently altered. The circulation of the fluid in an unhealthy state, produces a general debilitating effect: this is augmented by the fatigue resulting from the increased exertions which breathing requires. The imperfect expansion of the lungs causes in them a state of vascular congestion and consecutive serous effusion, which impedes the return of blood from the head, and gives rise to turgescence of the vessels of the brain with effusion into the cerebral cavities. 'Although,' says Dr. Cheyne, in his remarks on croup, 'apparently the first of the vital functions which is arrested be respiration, yet this seems to arise from a want of muscular strength in consequence of failure of the sensorial power, the invariable result of defective supply of pure arterial blood in the brain.' These patients, I believe, die more frequently from cerebral disorder and gradual exhaustion than from sudden or spasmodic suffocation." 147.

Our author questions the propriety of the term "spasmodic asthma," concluding that the dyspnœa observed in the paroxysm results from "vascular congestion of the lungs, and particularly of their mucous membrane." He acknowledges that when a permanent lesion exists, it is difficult to explain how the symptoms should be only occasional, the intervals of the attacks being quite free from suffering. Irregularity of the circulation, he thinks, offers a better solution of the difficulty than spasm.

"In whatever textures inflammation occurs, we notice periods of exacerbation and remission. May not, then, the occasional aggravation of the symptoms in laryngitis be as fairly ascribed to a temporary increase of the inflammation as to an extraordinary action of the muscles of the larynx? Are the distressing paroxysms in peritonitis ever attributed to spasm of the abdominal muscles? In tetanus, or in hydrophobia, where violent spasms of the throat are remarkable, do they ever produce asphyxia? To conclude these desultory remarks on spasm, I have only to say, with the late Dr. Albers, of Bremen, 'Neque adhuc vidi tracheitidem spasmodicam.'"* 154.

* Lib. cit. p. 153.

Several cases of the disease, as occurring in St. Bartholomew's and elsewhere, are detailed, but most of them have appeared in the periodicals at the time they took place. We come therefore to the second part of the paper.

### On Bronchotomy.

Obstruction to respiration, in cases of the above description, generally finds relief from bronchotomy; but where the obstruction is in the bronchial tubes, the operation is necessarily useless, if not worse. Whether bronchotomy may ever be serviceable in certain affections of the larynx, which have been termed spasmodic, cerebral, or sympathetic, our author does not pretend to determine, nor has he found any thing very satisfactory in his researches among written testimonies.

"Dr. Albers, of Bonn, speaks of a state of disease, observable particularly in girls who are subject to irregular menstruation, in which the larynx is so irritable, that, on the slightest occasion, and usually towards evening, it becomes affected with spasm, which is to be quieted by nothing, not even by musk and opium. It is attended with a dry cough, which will continue for two or three hours without interruption, and not cease until the patient falls into a swoon, or even into convulsions. 'I have seen,' says Dr. Albers, 'these symptoms regularly repeated every evening for two months together. In this case, as also in every instance where such spasm is produced, should not tracheotomy be employed to remove this troublesome and highly dangerous condition? Particularly as this affection continues to be regularly repeated even when the menstrual functions are performed, and as the patient is liable every moment to be suffocated."* 170.

Mr. Wood says he would prefer, in such cases, a trial of some powerful revulsive agent, such as sudden immersion in cold water, the shower-bath, croton-oil, or an emetic. The propriety of bronchotomy, in laryngeal disease, will much depend on the state of the lungs, which must be determined by an accurate investigation of the history and phenomena of the disease. We extract the following passage, which is founded on the authority of Bonetus, though we have great doubt of its accuracy.

"There are, perhaps, no symptoms that more frequently puzzle practitioners than those which seem to depend on affections of the larynx. Disease of the liver is sometimes attended with symptoms of cynanche laryngea, although no morbid appearances are presented in the larynx after death."† 173.

We verily believe that there is not in medicine a more widely extended error than this belief in cough and other affections really appertaining to the chest, being sympathetic of liver disease and dyspepsia. We have endeavoured, on many occasions, to point out the error, and the serious consequences thence resulting, but in vain. The evil appears on the increase rather than on the wane. As example is more potent than precept, we shall briefly allude to one or two melancholy cases illustrative of this point. There are some medical practitioners, not one hundred miles from Tunbridge, who can vouch for the truth of the following fact. A fine young gentleman became affected with cough, and apparently slight pul-

---

* Graefe and Walther's Journal, Bd. XV. Heft 4.

† Bonetus Sepulchretum, Tom. I. Lib. II. Sec. 1, Obs. 4.

monary affection. He brought up a little blood, and one of the most eminent of our metropolitan surgeons, happening to be in the neighbourhood, was consulted. He pronounced the complaint to be merely hepatic congestion, and ordered calomel, as a matter of course. The symptoms not giving way, the patient was brought up to town, when a very superficial examination of the chest shewed almost the whole of the left lung disorganized! He died a few weeks afterwards, and dissection proved the truth of the diagnosis. The family has been left in the greatest distress, and the false diagnosis of the surgeon has damaged his reputation through a very extensive circle of society.

This day (10th of January) we saw a young lady just arrived from the country, with "stomach-cough," of seventeen months standing. Not the slightest idea was entertained by some metropolitan and provincial physicians of any disease in the chest. Yet a pupil at any of our great hospitals might have discovered the existence of chronic inflammation of the lungs to an extent which now leaves little hopes of recovery. Mutton-chops for breakfast—meat and wine for dinner—bitters, nay tonics, were employed for many months to reduce the "stomach-cough"—with, we need hardly say, no benefit—but, on the contrary, with the loss of time, that can never be compensated for by any antiphlogistic measures! We could fill a whole number of our Journal with similar cases; but they would make no impression on the public—and "stomach-cough" will have its way.

Mr. Wood notices certain errors of diagnosis of a different kind.

"A child, eight months old, died with symptoms of cynanche laryngea, which were evidently occasioned by the pressure of an abscess in front of the thyroid cartilage, and bounding nearly the whole extent of the os hyoides. The internal lining of the air-passage was found healthy. The narrator of this case regrets, with much propriety, that the abscess was not discovered before death.

Mr. Lawrence has related the case of a young woman, under twenty years of age, who died suffocated from an aneurism behind the sternum, pressing on the trachea. Her symptoms were so deceptive that it was supposed bronchotomy would be required.

The sixth case detailed in the important Memoir of Bayle, is that of an aneurismal tumour, compressing the left bronchia, and simulating œdema of the glottis. 'I did not,' says Bayle, 'see this case, but am persuaded that I should have taken the disease for œdema of the glottis.'*

I know an instance where this mistake led to the performance of tracheotomy, which was attended with bursting of the aneurism into the trachea: and, from a preparation which I lately saw in the College of Surgeons, it appears probable that the same unexpected event must have annoyed another practitioner. In this preparation there is an artificial opening in the larynx; and just above the bifurcation of the trachea another opening is seen to communicate with the cavity of the ruptured sac of an aneurism.

Dr. Hope affirms, that bronchotomy has several times been performed with the view of obviating suffocation occasioned by aortic aneurism.†

It is unnecessary to multiply instances to prove the importance of minute diagnostic research. The obscurity in which the diseases of the larynx are often

---

* Nouveau Journal de Médecine, Janvier, 1819, p. 22.

† The Cyclopædia of Practical Medicine, p. 110.

involved, and the serious errors of practice to which an ill-founded opinion may lead, should induce us to investigate with great care every disturbance of respiration, especially where the source of suffering seems to be in the larynx. Before tracheotomy is undertaken the actual seat of the disorder must at least be determined. The co-existence of any important pulmonary lesion will of course considerably interfere with the prospect of a favourable issue. When we are not, however, to be deterred from operating either from the supposition or assurance that the lungs are simultaneously disordered with the larynx. If the cure of the disease were therefore out of the question, the operation, by lessening the frequent necessity of coughing to get rid of the increased mucous secretion, and the consequent fatigue to the lungs, at a time when their unhealthy condition requires all possible repose, might yet obtain for the patient alleviation of distress and prolongation of life." 176.

The general rule has been to have recourse to bronchotomy only in cases of extreme danger of suffocation, without reference to the benefit that might result from quietude of the parts, when in a state of disease. Mr. Lawrence has pointed out the advantages to be expected from an early operation—and the Germans claim the merit of proposing and performing it in chronic laryngitis, where there was no immediate danger of life. Mr. Wood considers that even ulceration is good cause why bronchotomy should not be performed. The author prefers the removal of a portion of cartilage to the introduction of a canula. This removal may sometimes be easy, where there is not much infiltration of the parts; but, we apprehend, that the canula will generally be necessary, not only at the time of operating, but for some time afterwards. Mr. Wood has searched for all information on the subject of bronchotomy; but as the result is a compilation of what was already known, we must pass it over. We therefore proceed to the third part of the paper.

### Wounds of the Throat.

These deserve to be considered among the causes of laryngeal inflammation and its serious consequences.

*Case.* "Thomas Tibbett, aged fourteen, having been detected in thieving, and roughly treated, cut his throat with a razor on the last day of January, 1831, and was immediately brought to St. Bartholomew's. There was a gaping transverse wound of the integuments about two inches in length. The windpipe was wounded in two places: one incision having been made through the upper part of the thyroid cartilage, and the other through the crico-thyroid membrane. He had lost very little blood, and no vessels required to be tied. The parts were approximated by keeping the head bent forwards, and no attempt was made to unite any part of the wound by ligature. As fluids, which he attempted to swallow, came freely out of the wound, inducing an opinion that the pharynx was injured,* and as deglutition excited cough, and was at-

* "That the escape of fluids through a wounded windpipe, during deglutition, is no sure criterion of a breach of structure of the gullet, is proved by two cases of self-murder recorded in the twelfth volume of the Lancet, and by the occasional occurrence of this symptom after the performance of bronchotomy for disease of the larynx."

tended with the inconvenience of separating the several cartilages, he was fed with milk by the aid of an elastic tube, passed through the nostril: to this operation he quietly submitted, as it was less troublesome than swallowing, and his appetite was keen. At first there was some frothy mucous discharge from the wound, with noisy and sibilant breathing; but the patient suffered little. On the 20th of February he still breathed through the opening, but did not require to be fed, and was able to walk about in the ward. When the prospect of recovery was most favourable, difficulty of breathing supervened. The upper wound of the larynx, which was much the larger one, being sufficient to allow a free entrance of air, the obstruction was considered to be caused by inflammation lower down in the windpipe, and leeches were accordingly applied. He died on the 27th of February.

The following appearances were found on dissection.

The lateral borders of the epiglottis were approximated; the membrane covering it and the neighbouring parts was swollen, and the glottis much contracted. The upper wound of the larynx just admits a large bougie. Its communication with the pharynx was nearly obliterated by cicatrization. The lower wound was healed, and scarcely recognizable externally, but clearly indicated within by a wrinkled and indented appearance of the lining membrane. Between the two wounds the chordæ vocales were involved in a fleshy swelling (èpaissisement par hypertrophie), through which a female catheter passed without difficulty. The tracheal membrane was rather more vascular than usual. There were extensive old adhesions of each pleura, with depositions of gritty and cheesy matter in the lungs and bronchial glands." 197.

It is probable, as Mr. W. observes, that this poor boy's life might have been preserved, had a tube been opportunely introduced through the wound, so as to traverse the seat of obstruction. Tracheotomy, also, might have effected the same object.

As it is not safe to draw inductions from individual cases, Mr. Wood has been at some pains to examine surgical records for instances bearing on the present subject. We shall introduce two or three abridged cases in illustration.

*Case* 1. "A workman, aged thirty-six, pursued for debt, and resolved to finish a life of trouble, cut his throat with a razor between the os hyoides and thyroid cartilage, and was conveyed to the hospital at Toulon on the 30th of Dec. 1819, four hours after the accident. The lips of the wound were brought together by adhesive straps, and a compress was applied; but ere long hemorrhage ensued, and it was necessary to tie three small arteries. The wound was again dressed, and the head inclined towards the chest. The next day the pulse was excited, but there was no other unfavourable symptom. Blood was taken from the arm, and soothing drinks were prescribed. On the fifth day alarming symptoms suddenly appeared; fits of imminent suffocation, agitation, heat of skin, paleness of face, tumefaction of the neck, complete loss of voice, hissing and very laborious inspiration, and less difficult expiration. A large bleeding was practised from the arm; fifteen leeches were applied on the neck, and sinapisms on the feet; but the paroxysms increased, and the patient died in the evening.

*Examination.* The upper part of the thyroid cartilage had been cut, and the hyo-thyroid membrane divided. There was considerable ecchymosis of the fold of membrane which unites the epiglottis to the arytænoid cartilage of the right side. The membrane of the epiglottis, and of the upper part of the larynx and its ventricles, was œdematous. The serosity was easily expressed from the swollen membrane. The lungs were the seat of a slight sanguineous infiltration."

*Case* 2. "A Swiss soldier made with a knife a triangular wound of his throat, about two inches long, at the upper border of the thyroid cartilage. He was immediately taken to the military hospital at Toulon. There was scarcely any bleeding: the wound was therefore at once dressed with strips of adhesive plaster, and covered with charpie and compresses, which were confined by a circular bandage. The patient was instructed to keep his head bent forwards. In four hours hemorrhage took place. When the dressings were removed, the blood flowed freely, but the bleeding orifices could not be found. A piece of agaric was applied, and the wound dressed (or rather smothered) as before. After an hour the charpie was again soaked in blood; but, as this did not trickle down the neck, the dressings were not disturbed.

A soft blueish swelling now formed at the base of the jaw on the left side. His breathing became very difficult, and the face injected; suffocation menaced; he frequently raised his hands to tear away the dressings. As the bandage seemed too tight, an ordinary chin-cloth was substituted. The wound ceased to bleed, and the patient felt relieved; but he continued restless and agitated, and often assumed the sitting posture, which seemed to give him ease. The difficulty of breathing soon increased; and he died six or seven hours after his entry into the hospital.

*Examination.* The course of the wound was occupied by clots of blood, which had accumulated in the situation of the swelling observed during life. All the cellular tissue of the anterior cervical region was infiltrated with blood, which had come principally from a wound of the laryngeal branch of the right superior thyroid artery. There was considerable œdema of the membrane investing the arytænoid cartilages; and the chink of the glottis was nearly obliterated by the swelling. The veins of the encephalon were more distended than natural, and the lungs were congested."

*Case* 3. "A young woman, who had divided the larynx just beneath the thyroid cartilage, went on well till the fourth week, when difficulty of breathing came on, and increased during twenty-four hours till death. The cavity of the windpipe above the wound was filled up by granulations of flesh: and when the external opening became so small that the lungs could not be supplied with air from that source, a difficulty of breathing commenced, and at length the patient was suffocated by the very efforts which Nature made to heal the divided parts." 203.

Some cases are next quoted from Dr. Rust, to which are appended some observations on the subject by the same author. These last, being concise, we shall give in the words of the German professor.

"The principal danger of such wounds is to be sought in the subsequent inflammation of the windpipe and lungs. Large doses of calomel, friction of mercurial ointment in the neighbouring parts, cold lotion to the neck, active local as well as general bleeding,—in a word, the reduction of life to its lowest pitch, are the most certain means to prevent this fatal inflammation. I was never fortunate enough to save any one thus wounded, unless where the powers had been almost exhausted by the accidental or prescribed loss of blood. All injuries of this sort have proved fatal when immediate assistance was afforded; and usually the quicker, as the loss of blood was smaller and the patient's strength promised a longer duration of life. Of the latter class, several unfortunate sufferers have died, when the windpipe has only been partially divided: whereas I have this day, in the hospital, perfected the cure of such a patient, even under the persistance of symptoms threatening danger, by means of venesection for three consecutive days, so as to produce fainting each time, together with leeches on the neck, large doses of calomel, and a spare diet.

The uniting of the divided windpipe by suture is not so essential as the ancients supposed, nor so objectionable as modern writers represent. Here, as in general, the middle way is the best, and a single loop appears as necessary in many cases to prevent the descent of the lower portion of the tube, as the employment of three or four sutures is superfluous and prejudicial.

Further observations and experiments must determine whether, as the above cases seem to teach, it is better, instead of immediately closing by sutures the external wound, to keep it open either totally or at least partially, until the wound of the windpipe and other deep parts is healed, so as to allow of free vent for any accumulation of pus or mucus." 216.

To the foregoing observations of Dr. Rust, Mr. Wood has appended some of his own. The whole paper is indicative of research and judgment. It was far better fitted for an article in the Cyclopædia of Practical Medicine, as an avowed compilation, than as an original article in the Transactions of the Medico-Chirurgical Society. It is doubtful whether the author may consider this as a compliment or not. If he be ambitious of the fame of originality, he will not thank us for the remark:—If he covet the more humble, but perhaps more useful, appellation of DILIGENT AND JUDICIOUS COMPILER, he will be conscious that he deserves our praise, quoad compilation, and possibly may be contented—a rare event in authorship!

